# Moulds and Mycotoxins in the Meat Production Chain from Slaughterhouse to Market: A Scoping Review on *Aspergillus* and *Penicillium* Isolation

**DOI:** 10.3390/foods15040630

**Published:** 2026-02-09

**Authors:** Melissa Alves Rodrigues, Lais Freitas, Letícia Estevinho, Claudemar Oliveira, Rosa Capita, Alexandra Esteves

**Affiliations:** 1 Doctoral School, University of León (ULE), Campus de Vegazana, E-24007 León, Spain; 2 Netherlands Food and Consumer Product Safety Authority (NVWA), P.O. Box 43006, 3540 AA Utrecht, The Netherlands; 3 Centro de Investigação de Montanha (CIMO), Instituto Politécnico de Bragança, Campus de Santa Apolónia, 5300-252 Bragança, Portugal; laisfreitas@ipb.pt (L.F.); leticia@ipb.pt (L.E.); m323203@alunos.ipb.pt (C.O.); 4 Laboratório para a Sustentabilidade e Tecnologia em Regiões de Montanha, Instituto Politécnico de Bragança, Campus de Santa Apolónia, 5300-253 Bragança, Portugal; 5 Department of Food Hygiene and Technology, Veterinary Faculty, University of León, E-24071 León, Spain; rosa.capita@unileon.es; 6 Institute of Food Science and Technology, University of León, E-24071 León, Spain; 7 Department of Veterinary Science, School of Agrarian and Veterinary Science (ECAV), University of Trás-os-Montes e Alto Douro (UTAD), 5000-801 Vila Real, Portugal; alexe@utad.pt; 8 Veterinary and Animal Research Center (CECAV), University of Trás-os-Montes e Alto Douro (UTAD), 5000-801 Vila Real, Portugal; 9 AL4AnimalS Associated Laboratory for Animal and Veterinary Science, 5000-801 Vila Real, Portugal

**Keywords:** *Aspergillus*, meat, meat products, mycotoxins, *Penicillium*

## Abstract

Fungal contamination of meat and meat products represents a significant concern for food safety, particularly due to the potential presence of mycotoxin-producing moulds. This scoping review aimed to map the occurrence and distribution of *Aspergillus* and *Penicillium* species along the meat production chain, from slaughterhouse environments to retail products, and to identify associated mycotoxins when reported. A systematic literature search was conducted in the PubMed database, complemented by a search in Google Scholar in accordance with Preferred Reporting Items for Systematic Reviews for Scoping Reviews (PRISMA-ScR) guidelines. Eligible studies reported the isolation of *Aspergillus* and/or *Penicillium* species from meat, meat products, or meat-processing environments under natural contamination conditions. The results indicate that both genera are frequently detected throughout the production chain, particularly at processing and storage stages, with several studies reporting species known for mycotoxin production. In addition, the presence of these moulds in processing environments highlights potential implications for both food safety and occupational exposure. However, information on mould occurrence in meat, edible offal, meat products and meat processing environments remains scarce, fragmented and heterogeneous. Overall, this review highlights existing knowledge gaps and underscores the need for harmonised monitoring strategies and further research addressing fungal contamination and mycotoxin risks along the meat production chain.

## 1. Introduction

Moulds, including species belonging to the *Aspergillus* and *Penicillium* genera, are well known to cause spoilage in food products. Meat and meat products are among the main foodstuffs that may deteriorate under their influence [1]. Fungal cells can be found in the air, on surfaces, in water, on equipment, in food manufacturing ingredients, and on workers [1,2]. Although some mould species may exert protective effects against undesirable microorganisms, others can produce toxic secondary metabolites [1].

Mycotoxins are secondary metabolites mainly produced by filamentous fungi and are considered an increasing health hazard, as some of them may cause organ damage, immunosuppression, certain types of cancer, or death [3]. Mycotoxin contamination of meat and meat products has been documented to occur through different routes, namely: (i) carry-over from animals exposed to contaminated feed; (ii) contamination originating from the environment; and (iii) contamination via spices and raw materials used during the processing phase [4].

Environmental and technological factors, such as temperature (10–45 °C), pH (1.5–10), and water activity (≥0.6), strongly influence the occurrence and growth of moulds in meat and meat products [2]. *Aspergillus* and *Penicillium* species, which can grow under a wide range of environmental conditions, are frequently associated with mycotoxin production in meat products [5,6] and have been identified at different stages and settings of the meat chain.

Several *Aspergillus* and *Penicillium* species are known to produce a wide range of mycotoxins [7]. In meat products, *A. flavus*, *A. niger*, *A. ochraceus*, *A. parasiticus*, *A. steynii*, *A. subramaniannii*, *A. versicolor*, and *A. westerdijkiae* are among the mycotoxin-producing moulds that have been isolated. In addition, numerous *Penicillium* spp. have also been identified, including *P. aurantiogriseum*, *P. brevicompactum*, *P. chrysogenum*, *P. citrinum*, *P. commune*, *P. crustosum*, *P. cyclopium*, *P. expansum*, *P. glabrum*, *P. griseofalvum*, *P. nordicum*, *P. oxalicum*, *P. palitans*, *P. polonicum*, *P. roquefortii*, *P. rugulosum*, *P. verrucosum*, and *P. viridicatum* [6].

The Commission Regulation (EU) 2023/915 [8] establishes, among other contaminants, maximum levels of mycotoxins in foodstuffs in Annexe I. However, no maximum limits for mycotoxins in meat and meat products are currently set. Among the most toxic mycotoxins reported in meat, aflatoxin B1 (AFB_1_) and ochratoxin A (OTA) are the most frequently detected [4]. Between 1990 and 2025, several studies have been reported the detection of mycotoxins, including in offal samples collected from slaughterhouses in Mozambique [9]; in meat products and raw materials from meat processing facilities in Croatia [10], Italy [11], Egypt [12], Portugal [13], and Spain [14]; and in meat products purchased in Croatia [15,16], Cyprus [17], and Italy [18]. A detailed overview is provided in Supplementary Table S1.

Aflatoxins, which are classified as carcinogenic and genotoxic compounds, are mainly produced by *A. flavus* and *A. parasiticus* [19]. Between 1990 and 2025, aflatoxins have been reported in poultry livers and gizzards sampled in slaughterhouses in Mozambique [9], in spices used in meat processing [12] and in meat products sampled in meat processing facilities and markets [12,15,17].

Aflatoxin B_1_ (AFB_1_), the most frequently detected aflatoxin in food, is classified as a Group 1 carcinogen by the International Agency for Research on Cancer (IARC) [3,20]. After ingested, AFB_1_ is metabolised in the liver into several reactive metabolites, including aflatoxin-8,9-epoxide, which can bind to cellular proteins, causing acute toxicity, or form adducts with DNA, leading to hepatocarcinogenesis. In addition, AFB_1_ metabolism generates reactive oxygen species and produces aflatoxin M_1_ (AFM_1_), a hydroxylated metabolite that retains toxicological properties comparable to those of AFB_1_ [9]. It has been recommended that foods intended for human consumption should not contain more than 10 μg/kg of total aflatoxins, with AFB_1_ not exceeding 5 μg/kg [3].

Ochratoxin A (OTA), another major contaminant of meat products, is classified as a Group 2B carcinogen by IARC and is produced by several mould species belonging to both the *Aspergillus* and the *Penicillium* genera [4]. OTA is frequently detected in the kidneys of slaughtered animals, and pork consumption, particularly in Eastern Europe, has historically been an important source of human exposure. Due to its ability to bioaccumulate in monogastric species, OTA is commonly found in edible pig tissues and pork products [4]. In the meat industry, *A. ochraceus*, *P. nordicum*, and *P. verrucosum* are among the most common producers of OTA [7,19]. Although no European Union (EU)-wide legal limits have been established for OTA in meat, several European countries have previously established national maximum levels. Available literature indicates that Denmark, Estonia, Romania and Slovakia had set maximum permitted OTA concentrations, while Italy had developed national guidelines for recommended maximum OTA levels [19,21]. More recently, in Italy, the Sezione Sicurezza Alimentare—Comitato Nazionale per la Sicurezza Alimentare (CNSA) issued an opinion on May 2021, reinforcing the reference level of 1 µg/kg for OTA in pork products, as initially established by Circular No. 10 from the Ministry of Health, dated 9 June 1999 [22].

Several studies have demonstrated that specific conditions, such as temperature, relative humidity, pH, and water activity (a_w_), can promote the uncontrolled growth of moulds, including *Aspergillus* and *Penicillium*. In addition, inadequate environmental hygiene and suboptimal production practices can further enhance mould proliferation and the production of the aforementioned mycotoxins in meat and meat products [4]. Furthermore, certain species, such as *A. fumigatus*, as well as chronic exposure to mycotoxins through inhalation and dermal exposure to mycotoxins (e.g., AFB_1_), may represent occupational health hazards for workers along the meat production chain [2].

Although several reviews have addressed fungal contamination of meat and meat products, none have comprehensively examined the occurrence of fungal species across different stages of production and marketing, including both product and environmental samples. This review aims to address this gap by providing an overview of *Aspergillus* and *Penicillium* species, particularly those capable of producing mycotoxins that may contaminate the environment, meat, and meat products in slaughterhouses, meat processing facilities, and meat markets. Specifically, this review seeks to identify which species are associated with contamination of these facilities, discuss the potential implications for occupational health and food safety, evaluate the risk of mycotoxin production based on the isolated species, and highlight gaps in the literature regarding the occurrence and mycotoxigenic potential of these fungi in meat and related environments.

## 2. Materials and Methods

### 2.1. Database Search Strategy

The study aimed to conduct a scoping review of the available literature on the isolation of fungi belonging to the genera *Aspergillus* and *Penicillium* along the meat production chain (slaughterhouses, meat processing facilities, and retail markets). The search protocol was in line with the Preferred Reporting Items for Systematic Reviews for Scoping Reviews (PRISMA-ScR) guidelines [23].

Between 7th October and 18th November 2025, a structured literature search was conducted in the PubMed database, complemented by a Google Scholar search by one reviewer. Studies published between 1990 and 2025 were considered eligible. Limitations related to the database search strategy are acknowledged and discussed in the Section 3.5.

The search strategy was designed to broadly capture the presence of fungi and mycotoxins along the meat production chain of food-producing animals. The following Boolean search strings were used at the PubMed database: (a) *Aspergillus* AND meat OR meat products; (b) fungi AND farm AND feed; (c) fungi AND meat; (d) fungi AND meat processing plants; (e) fungi AND meat products; (f) moulds AND animal feed; (g) moulds AND animals; (h) moulds AND bovine health: (i) moulds AND bovine AND abortion; (j) moulds AND bovine AND respiratory; (k) moulds AND bovine AND aspergillosis; (l) moulds AND poultry; (m) moulds AND swine; (n) moulds AND sheep; (o) moulds AND meat; (p) moulds AND slaughterhouse; (q) moulds AND meat market: (r) (moulds AND meat) OR (moulds AND slaughterhouses); (s) *Penicillium* AND meat OR meat products; (t) yeasts AND slaughterhouse.

The following Boolean search strings were used at the Google Scholar database: (a) *Aspergillus* AND meat OR meat products; (b) moulds OR yeasts AND meat; (c) moulds OR yeasts AND slaughterhouses; (d) *Penicillium* AND meat OR meat products.

A total of 3374 records were initially screened by title and abstract. Of these, 3183 records were excluded because they were not within the scope of this review. Full texts of 191 articles were assessed for retrieval.

### 2.2. Eligibility Criteria

Of the articles selected for eligibility assessment, 123 were excluded for one or more of the following reasons:

(i) written in a language other than English, Spanish, Portuguese or Dutch;
(ii) full text unavailable or no longer accessible;
(iii) not original research articles or not yet accepted for publication;
(iv) the study applied artificial inoculation of fungi;
(v) use of fungal isolates from previous studies or culture collections (e.g., focusing only on secondary analyses, such as mycotoxin production) without reporting original occurrence data from the meat production chain;
(vi) fails to specify the fungal species isolated;
(vii) focused solely on mycotoxin occurrence due to a lack of fungal isolation data;
(viii) unclear sampling location (not specified if the sampling occurred specifically in the slaughterhouse, meat processing facilities, or market).

### 2.3. Study Selection and Data Extraction

The first phase aimed to collect all data on fungal species isolated at the stages of the meat production chain mentioned. However, due to heterogeneity across studies in methodology and reporting formats, the analysis was refined into a scoping review of the isolation of filamentous fungi in slaughterhouses, meat processing facilities, and meat markets, focusing on *Aspergillus* and *Penicillium*, given their relevance as mycotoxin-producing genera and potential impact on human health.

The initial data collection, based on 68 articles, focused on all isolated fungal species (moulds and yeasts), recording the sampling site, the type of facility, the sort of meat or meat products processed on that facility (e.g., in slaughterhouses it was registered the slaughtered animal species), the number of isolates of each fungal species, the percentage frequency of isolates, the number of positive samples, the percentage frequency of positive samples, the types of samples from which the species were isolated, the country where the study was conducted, and the corresponding reference.

After this, based on that database, only species belonging to the genera *Aspergillus* and *Penicillium* were selected. A total of 16 articles were excluded at this stage for not reporting the isolation of *Aspergillus* spp. and/or *Penicillium* spp. Species reported in the original studies under outdated nomenclature but currently assigned to these genera were not included, as current taxonomic assignments were not systematically verified. This approach provided a clear and reproducible framework for species inclusion, focusing on species with clearly identifiable names within the *Aspergillus* and *Penicillium* genera, although some data may have been excluded. This limitation is acknowledged and discussed in the Section 3.5.

Given the wide variability in identification and quantification methodologies across studies and the lack of consistency in how results were presented, it was decided that only the isolated *Aspergillus* and *Penicillium* species would be reported, along with the specific facilities, types of facilities, products, and countries in which they were detected, to provide an overall perspective on the distribution of these species within the mentioned settings. Thus, information on the absolute and relative frequency of isolates or positive samples was not included.

The charted data were then summarised descriptively, using tables to highlight distributions across production stages and sample types. As mentioned, no quantitative synthesis was performed due to heterogeneity in study designs, sampling methods, and reporting practices. Moreover, no in-depth assessment of the quality of the included studies was conducted. The primary aim was to map the extent and nature of the available evidence rather than to evaluate its methodological rigour, since the considerable heterogeneity among studies regarding sampling approaches, identification methods, and reporting practices would have limited the applicability and meaningfulness of a formal quality assessment. Following data analysis, this review protocol was registered in the Open Science Framework (OSF) Registries (https://doi.org/10.17605/OSF.IO/9SCDG).

## 3. Results and Discussion

### 3.1. Literature Search Results

**Figure 1:**
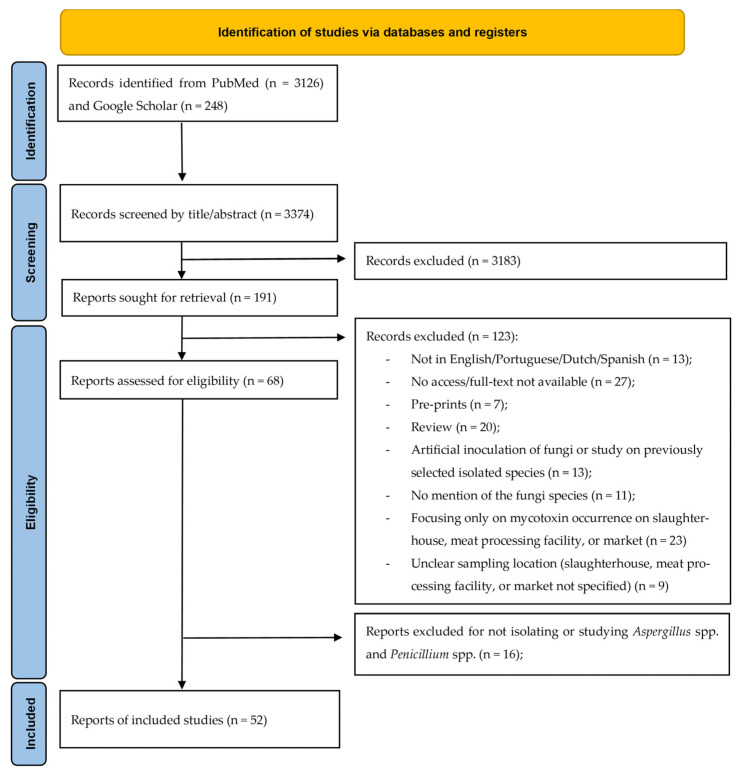
PRISMA-ScR flowchart of literature search, inclusion and exclusion criteria, and data collection.

The literature search yielded 3374 records. After application of the predefined eligibility criteria, 52 studies were included in the scoping review for further analysis. An overview of the selection process is presented in Figure 1. The included studies, conducted between 1990 and 2025, originated from 24 geographical regions and were predominantly observational.

The included studies were carried out in Argentina (*n* = 2) [24,25]; Austria (*n* = 2) [26,27]; Brazil (*n* = 3) [28,29,30]; China (*n* = 1) [31]; Croatia (*n* = 3) [10,15,32]; Denmark (*n* = 1) [33]; Egypt (*n* = 8) [12,34,35,36,37,38,39,40]; Greece (*n* = 1) [41]; India (*n* = 1) [42]; Iraq (*n* = 3) [43,44,45]; Italy (*n* = 7) [11,26,46,47,48,49,50]; Japan (*n* = 1) [51]; Korea (*n* = 2) [52,53]; Nigeria (*n* = 4) [54,55,56,57]; Norway (*n* = 2) [58,59]; Pakistan (*n* = 1) [60]; Portugal (*n* = 2) [13,61]; Saudi Arabia (*n* = 3) [38,62,63]; Serbia (*n* = 1) [64]; Slovakia (*n* = 1) [65]; Slovenia (*n* = 1) [66]; Spain (*n* = 2) [67,68]; Sweden (*n* = 1) [69]; and Turkey (*n* = 1) [70]. Both Peintner et al. [26] and Aljazzar et al. [38] presented data from more than one country within a single study.

Seventeen studies were conducted in the slaughterhouse, sampling air, equipment, meat, workers’ nasal swabs, water, surfaces, and tools (Table S2, Supplementary Materials). Twenty-four studies reported the isolation of *Aspergillus* spp. and/or *Penicillium* spp. in meat processing facilities, mainly from air and meat products (Table S3, Supplementary Materials. Fourteen studies focused on retail meat markets and sampled meat and meat products (Table S4, Supplementary Materials).

Based on the studies included in the scoping review, the occurrence of moulds along the meat production chain was analysed by genus, starting with *Aspergillus* spp., due to their high prevalence and clinical relevance.

### 3.2. Aspergillus spp. Isolation Across the Meat Production Chain

Fungi isolated from meat production settings may represent both potential meat-borne pathogens and occupational hazards [2]. *Aspergillus* species, which are among the leading causes of human mould infections, can affect the health of both immunocompetent and immunocompromised individuals, causing hypersensitivity reactions, chronic pulmonary infections, and, in severe cases, life-threatening invasive disease infections [2,71]. Following inhalation or direct inoculation of *Aspergillus* conidia, infection may develop locally or disseminate systemically [72].

In addition to their clinical relevance, several *Aspergillus* species are able to produce mycotoxins, such as AFB_1_ and OTA [61]. *Aspergillus flavus* is a major producer of AFB_1_, and high occupational exposure risks associated with inhalation of this toxin have been reported for other mycotoxins [61]. Occupational exposure to AFB_1_ among poultry slaughterhouse workers has been documented and may occur through inhalation or dermal absorption [73]. However, studies specifically assessing occupational exposure to mycotoxins in meat industry environments remain limited.

*Aspergillus fumigatus*, followed by *A. flavus*, *A. terreus*, and *A. niger*, is of particular clinical relevance, being considered the primary etiologic agent of invasive aspergillosis [2,61]. This species is also capable of producing immunosuppressive secondary metabolites, such as gliotoxin, and has been associated with respiratory symptoms including asthma, allergic sinusitis, cough, and bronchial hyperresponsiveness [2].

Beyond occupational exposure, *Aspergillus* spp. may colonise edible organs, meat, and meat products, contributing to mycotoxin production under favourable conditions [37,56,74,75,76]. The production of AFB_1_ by *A. flavus* and *A. parasiticus* and of OTA by *A. ochraceus* and *A. westerdijkiae* on dry-cured meat products has been confirmed. In addition, sterigmatocystin production by *A. versicolor* has been reported [6]. Other mycotoxins, including aflatoxin B_2_, aflatoxin G_1_, aflatoxin G_2_, cyclopiazonic acid, 3-nitropropionic acid, fumonisin B_2_, penicillic acid, xanthomegnin, viomellein, and vioxanthin, have also been produced by *Aspergillus* spp. isolated from meat products and spices used in meat processing [12,13,19,77,78]. The most common mycotoxin-producing *Aspergillus* spp. previously detected on meat products are presented in Table 1.

Mycotoxin production in contaminated meat products is influenced by factors such as a_w_ and temperature. The production of AFB_1_ by *A. flavus* and *A. parasiticus*, for instance, is favoured at a_w_ ≥ 0.80 and ≥0.84, respectively, at a temperature range of 12 to 35 °C [80]. *Aspergillus ochraceus* is reported to produce OTA at a_w_ ≥ 0.87 and under 12–35 °C [80].

The main *Aspergillus* species isolated in slaughterhouses, meat processing facilities, and meat markets are presented in Table 2. An overview of the remaining *Aspergillus* species isolated is presented in Table S5 (Supplementary Materials).

To better characterise the distribution of *Aspergillus* spp., results are presented according to the main contamination routes, including air, surfaces and equipment, and food matrices.

#### 3.2.1. *Aspergillus* spp. Isolated from the Air and Nasal Swabs

*Aspergillus flavus*, *A. fumigatus*, *A. niger*, *A. sydowii*, *A. terreus*, and *A. versicolor* are among the *Aspergillus* species detected in the air in different sorts of slaughterhouses (Table 2). Moreover, *A. fumigatus*, *A. niger*, *A. sydowii*, and *A. versicolor* were also isolated from the production environment (including air) of a meat production facility (Table 2).

From an occupational health perspective, exposure to bioaerosols, containing airborne fungi and mycotoxins, represents a health risk [61]. Notably, *A. flavus* and *A. niger*, among the *Aspergillus* species with mycotoxigenic and clinical relevance, have also been detected in nasal swabs collected from slaughterhouse workers (Table 2), highlighting inhalation as a relevant route of exposure. Seasonal variation, temperature, relative humidity, building materials, facility age, and ventilation conditions significantly influence fungal concentrations and species diversity in indoor environments [2,59].

High airborne spore loads may also contribute to secondary contamination and growth of toxigenic fungi on meat and meat products [59]. Facility design and layout influence air circulation patterns and may promote airborne dissemination of fungal spores onto products and contact surfaces [2,59]. The adoption of standardised sampling protocols and complementary identification methods would improve the comparability and reliability of *Aspergillus* spp. prevalence data in these environments.

#### 3.2.2. *Aspergillus* spp. Isolated from Surfaces, Equipment, Facilities, Tools, and Vectors

*Aspergillus sydowii*, *A. terreus*, *A. versicolor*, *A. flavus*, *A. fumigatus*, and *A. niger* are reported across multiple studies on surfaces, including floors and walls, in slaughterhouse environments (Table 2). Additional *Aspergillus* species were isolated from equipment, facilities, and tools (Table S5). Since contamination and growth of mycotoxigenic fungi may result from inadequate hygiene of rooms and equipment [59], these findings underscore the importance of effective cleaning and disinfection practices.

The implementation of regular and standardised fungal monitoring programmes for surfaces, integrated into Standard Sanitary Operating Procedures (SSOPs), is essential to enhance food safety [2]. Moreover, *A. flavus*, *A. fumigatus*, *A. parasiticus*, *A. niger*, and *A. ochraceus* were isolated from houseflies in slaughterhouse settings (Table 2), highlighting the role of vectors in fungal dissemination and reinforcing the need for effective pest control measures.

#### 3.2.3. *Aspergillus* spp. Isolated from Offal, Meat, Meat Products and Raw Materials

Several *Aspergillus* species, including *A. flavus*, *A. fumigatus*, *A. niger*, *A. ochraceus*, *A. terreus*, and *A. versicolor*, were isolated from offal samples (Table 2). Furthermore, carcass samples and meat swabs yielded *A. flavus*, *A. fumigatus*, *A. niger*, *A. ochraceus*, *A. sydowii*, *A. terreus*, and *A. versicolor* (Table 2). However, these species are not consistently reported across studies, likely because few investigations include sampling of meat swabs and offal. Consequently, although a range of *Aspergillus* species has been identified, only a limited number of studies report them in these sample types.

In meat products sampled at the processing stage, a broader diversity of species was reported, including *A. nomius*, *A. parasiticus*, *A. tamarii*, and *A. westerdijkiae* (Table 2). The presence of mycotoxigenic fungi on meat and meat products may result from airborne contamination, contaminated raw materials, and inadequate hygienic handling and processing conditions [2,59]. Mechanical damage to products, such as cracks in dry-cured meat products, creates favourable microclimatic conditions for mould growth. Therefore, producers must ensure adequate equipment performance and optimised processing conditions [59]. In addition, fungal contamination of animals at slaughter has been suggested as a potential source of cross-contamination within slaughterhouse environments [30].

*Aspergillus flavus* and *A. niger* were also detected in samples of spices used in meat processing (Table 2). Although many spices possess antifungal properties, they can also be contaminated by moulds. Species from the genera *Aspergillus* and *Penicillium* are among the most common contaminants [19]. Under poor storage and processing conditions, spices such as black pepper and nutmeg may become contaminated with aflatoxins and OTA [19].

In addition to Good Manufacturing Practices (GMP) and hygiene control, the application of biocontrol agents has been proposed as a promising strategy to limit the growth of mycotoxigenic *Aspergillus* species in dry-cured meat products [6]. *Debaryomyces hansenii* has demonstrated antifungal activity against *A. parasiticus* and *A. westerdijkiae*, while bacterial species such as *Staphylococcus xylosus* have shown inhibitory effects against *A. flavus*, *A. parasiticus*, and *A. westerdijkiae*, as well as against the production of AFB_1_ and OTA [6].

### 3.3. Penicillium spp. Isolation Across the Meat Production Chain

The growth of toxigenic moulds is favoured in environments with high relative humidity and moderated temperatures [6]. *Penicillium* spp. are among the most common filamentous fungi in the food processing industry, proliferating at lower to mid temperatures and tolerating low a_w_ (0.78–0.83) [2,19].

Certain *Penicillium* species, such as *P. nalgiovense* and *P. salamii*, which have been used as starter cultures, are beneficial in meat processing, especially during ripening. They can enhance the sensory qualities of the product and form a protective biofilm on the casing of specific meat products, inhibiting undesirable microorganisms [1]. However, spoilage species such as *P. nordicum* or *P. verrucosum*, found in different meat products, can produce mycotoxins (Table 3).

As previously mentioned, a_w_ and temperature affect mycotoxin production in meat products. It has been reported that the production of OTA by *P. verrucosum* is favoured at a_w_ ≥ 0.85 and at a temperature range of 2 to 34 °C [80]. On the other hand, it has been documented that *P. commune* produces cyclopiazonic acid over a range of aw ≥ 0.90 and at 12–30 °C [80].

The main *Penicillium* species isolated in slaughterhouses, meat processing facilities, and meat markets are presented in Table 4. An overview of the remaining *Penicillium* species isolated is presented in Table S6 (Supplementary Materials).

#### 3.3.1. *Penicillium* spp. Isolated from the Environment and Vectors

*Penicillium aurantiogriseum*, *P. brevicompactum*, *P. chrysogenum*, *P. citrinum*, *P. commune*, *P. crustosum*, *P. expansum*, *P. nordicum*, *P. polonicum*, and *P. roqueforti* are among the *Penicillium* species which have been reported in the air and surface samples collected in slaughterhouses, as well as in the environment (including air) of meat processing facilities (Table 4). Furthermore, *P. aurantiogriseum* and *P. verrucosum* were previously isolated from houseflies in the slaughterhouse setting (Table 4).

The findings underscore the fact that mycotoxigenic *Penicillium* species may spread in the environment through the air, surfaces, and vectors, representing a potential source of contamination of offal, carcasses, meat, and meat samples.

Microorganisms such as airborne fungi may be easily carried through the air in slaughterhouses that do not provide adequate separation between dirty or outdoor areas and clean areas [56]. In addition, Alapont et al. [68] have already concluded that the air in the ripening chambers of dry-cured ham is an important source of *Penicillium* contamination. In meat processing plants, as the air pressure gradient in the sorting room is higher, there is a chance of migration of aerosolised spores to the neighbouring production rooms, so it is important to adjust the air pressure gradients and guarantee a clear segregation between clean and unclean areas [59]. Furthermore, in order to decrease the spore concentration in the air, it is recommended to improve air circulation [59].

Moreover, moulds have been isolated from the walls and ceilings of slaughterhouses, salting, brining, and washing rooms, as well as from production materials such as sticks, nets, towels, brushes, and trucks, which harboured spores of the associated mycobiota [59], reinforcing the importance of the sanitization and disinfection of the facilities and tools. Additionally, it is highly recommended to implement strict vector control protocols in order to prevent cross-contamination.

#### 3.3.2. *Penicillium* spp. Isolated from Meat and Meat Products

Across the studied meat chain settings, *P. aurantiogriseum*, *P. chrysogenum*, *P. citrinum*, and *P. nordicum* have been identified in meat samples, including carcasses (Table 4). *Penicillium* spp. presence on these samples may result from environmental contamination, as previously discussed, and inadequate sanitation [40,56].

Additionally, *P. aurantiogriseum*, *P. brevicompactum*, *P. chrysogenum*, *P. citrinum*, *P. commune*, *P. crustosum*, *P. italicum*, *P. expansum*, *P. nordicum*, *P. polonicum*, *P. roqueforti*, and *P. verrucosum* have also been isolated from meat product samples (Table 4).

Numerous studies have shown that several factors can allow uncontrolled mould growth on dry-cured meat surfaces. These factors include specific temperatures, pH values, and water activity, as well as physical characteristics such as casing cracks, the presence or absence of a crust (as in prosciuttos), or insufficient washing and brushing. Such conditions can promote the production of mycotoxins by superficial *Penicillium* species. This route of contamination is considered significant in dry-cured meat products [4].

In meat products, the addition of salts, such as NaCl, lowers a_w_ [76]. Many of the isolated *Penicillium* species tolerate low a_w_, and several *Penicillium* species found, such as *P. brevicompactum*, *P. citrinum*, *P. nordicum*, and *P. polonicum*, are known to grow well even in the presence of 5% NaCl [76].

Also, the temperature used for ripening influences mould growth and their ability to produce mycotoxins [6]. As previously noted, *Penicillium* spp. proliferate at low to mid temperatures. For instance, *P. nordicum* can grow at temperatures between 10 and 25 °C [80]. Furthermore, since traditional meat products sold in the markets and fairs are often prepared in households under highly inconsistent and uncontrolled conditions, the occurrence and diversity of isolated moulds may be linked to the climatic conditions of the production region [15].

Moreover, the application of spices during meat processing may represent a contamination stage. Spices are mainly imported from developing countries with tropical and subtropical climates, where high temperatures, heavy rainfall, and humidity often promote fungal growth and increase the risk of mycotoxin contamination. As previously mentioned, some spices are particularly susceptible to contamination by toxigenic moulds, with *Aspergillus* and *Penicillium* being the most common contaminants. Furthermore, spices are often left to dry on the ground in open-air conditions, and poor outdoor hygiene further enhances mould growth and mycotoxin production [4].

Considering the findings presented, the importance of maintaining strict hygienic conditions in these environments is evident, as they play a key role in preventing and controlling the contamination and spread of *Penicillium* spp. Good hygiene practices, along with effective cleaning, disinfection, and monitoring protocols, are essential to minimise fungal proliferation and ensure safer meat production [2]. Additionally, proper control of ingredients used in meat product formulation should not be overlooked [20], nor should the implementation of effective vector control programmes.

Furthermore, some native microorganisms, such as specific yeast species, act as biocontrol agents (alone or in combination) against some *Penicillium* spp. *Debaryomyces hansenii* has been described as possessing antifungal activity to reduce the growth of *P. nordicum* and *P. verrucosum*. Other yeasts and bacterial species, such as *Candida zeynaloides* and *Staphylococcus xylosus*, have also been evaluated for their effects on *P. nordicum* growth and OTA production in dry-cured meat products [6]. Also, the antifungal properties of various plant extracts, particularly essential oils, are being investigated for use in meat and meat products, although their practical application in the meat industry remains limited and underdeveloped [6].

### 3.4. Risk Assessment of Fungal Contamination and Mycotoxin Exposure in Meat Production

Most of the relevant *Aspergillus* and *Penicillium* species isolated from the meat production chain environments reviewed appear to be globally ubiquitous. The greatest diversity of these genera is observed during the meat processing stage. Certain species, such as *A. nomius*, *A. westerdijkiae*, *P. cyclopium*, *P. nordicum*, *P. palitans*, and *P. rugulosum* were exclusively isolated from meat processing plants (Table 2 and Table 4). However, the higher number of studies reporting *Aspergillus* and *Penicillium* spp. in processing plants likely reflects a research gap in slaughterhouses and retail environments rather than true differences in contamination levels. Nevertheless, it is suggested that the processing stage is particularly critical. It combines conditions that favour fungal growth and mycotoxin production, and it may also introduce additional contamination through raw materials, such as spices. Although subsequent processing steps may reduce fungal contamination in slaughterhouses and processing plants, products at the market stage are already available for consumption, leaving limited opportunities for intervention.

Despite the lack of comparative studies across all stages of the meat production and distribution chain, occupational exposure is hypothesised to be highest in slaughterhouses, followed by meat processing plants. This assumption is supported by the elevated levels of airborne dust and direct contact with contaminated tissues, particularly in lairage areas and during bleeding or evisceration. However, this conclusion remains to be confirmed scientifically. The humid environment and the presence of high levels of organic substrates in slaughterhouses further promote fungal growth [2]. In meat processing plants, ripening chambers may also represent an exposure risk, although data on this are limited.

Mycotoxins occur naturally in spices, particularly susceptible to contamination by mycotoxigenic fungi [81]. During handling and processing, dry products, such as spices, can release mould spores and mycotoxins [82], creating an additional route of occupational exposure through inhalation of contaminated dust. Airborne OTA concentrations have been reported to range from <0.003 to 1.45 ng/m^3^ during nutmeg processing, and from <0.003 ng/m^3^ to 8.15 ng/m^3^ during black pepper processing [83]. Airborne OTA has also been detected in environmental and personal samples from workplaces where spice blends are used to prepare meat sausages [84]. These findings suggest that spices and other non-meat ingredients may contribute to environmental contamination by fungal spores and mycotoxins in meat processing plants.

Exposure to mycotoxins can occur via inhalation, dermal absorption, and ingestion. Importantly, some studies suggest that inhalation exposure may be more relevant for health effects than dietary intake [73]. Thus, it can be inferred that occupational exposure in meat production settings can lead to high localised exposure over time, potentially exceeding dietary intake from meat, as it may involve both inhalation and dermal absorption of mycotoxins.

In terms of dietary exposure from meat consumption, the latest European Food Safety Authority (EFSA) Scientific Opinion on OTA identified preserved meat as an important contributor to chronic intake, particularly pork ham, preserved meats, and sausages [85]. However, data on mycotoxin occurrence in meat and meat products remain limited, and no specific maximum limits for OTA or other mycotoxins are currently established in EU legislation [6]. According to the Food and Agriculture Organisation of the United Nations (FAO) database on worldwide mycotoxin regulations [86], which requires updating, some EU Member States (Denmark, Estonia, Italy, Romania, and Slovakia) had previously set national limits for mycotoxins in meat, as already mentioned. Similar regulations were also set in non-EU countries such as Serbia, Montenegro, and Ukraine. Reported maximum levels ranged from 0.5 to 5 µg/kg for AFB_1_ and from 1 µg/kg to 20 µg/kg for OTA. Harmonised regulatory limits for mycotoxins in meat and meat products are still lacking.

To strengthen risk assessment, research should prioritise linking fungi detected in the meat production environment with mycotoxin levels in products. Dietary exposure should be evaluated by comparing measured mycotoxin concentrations with reference values, such as tolerable daily intake. Occupational exposure should be assessed by integrating airborne mycotoxin and fungal data with epidemiological and biomonitoring information.

Regular and standardised fungal monitoring of air and surfaces, integration of mould control into sanitation programmes, pest control, and the control of environmental parameters are also essential. Although routine monitoring may be impractical in small-scale slaughterhouses and meat processing facilities, sporadic analyses should be encouraged. It should be noted, however, that assessing the risk of mycotoxin contamination based solely on fungal isolation is unreliable, as mycotoxins can persist in products and environment even in the absence of detectable fungal growth [2]. Moreover, not all isolated mycotoxigenic fungi will necessarily produce toxins, as mycotoxin formation strongly depends on environmental and substrate factors such as temperature, water activity, and pH.

Producers should implement Hazard Analysis and Critical Control Points (HACCP)-based programmes to reduce fungal and mycotoxin contamination. Key measures include controlling room temperature, removing excessive surface mould from products, ensuring adequate airflow, maintaining effective sanitation [4] and the use of certified, high-quality raw materials. Proper storage and handling of raw materials are also recommended to minimise environmental contamination. Furthermore, to reduce occupational exposure and food contamination, preventive measures such as hand hygiene, the use of personal protective equipment, and worker training and awareness programmes should be implemented [2].

### 3.5. Knowledge Gaps and Review Limitations

Overall, few studies have assessed the isolation of *Aspergillus* and *Penicillium* spp. and their associated mycotoxins in fresh meat. In addition, there is a lack of studies evaluating the presence and concentrations of moulds, including *Aspergillus* and *Penicillium*, and their mycotoxins in air and on surfaces of cold stores and transport vehicles, and how this relates to contamination of products sold in markets and supermarkets. Similarly, there is limited research on the economic impact of isolating mycotoxigenic *Aspergillus* and *Penicillium* throughout the meat production chain. Moreover, a key research gap is the lack of studies linking environmental fungal loads to contamination levels in meat and meat products. This leaves open the question of how effectively product contamination can be prevented by controlling specific environmental parameters. It also remains unclear which parameters should be prioritised for monitoring and control.

In slaughterhouses, studies tracing the occurrence of moulds, including *Aspergillus* and *Penicillium* spp., carried by slaughtered animals on meat and edible offal, and their impact on carcass and offal rejection, are scarce. There is also a lack of data assessing the occupational burden posed by *Aspergillus* and *Penicillium*, and their mycotoxins, in these facilities, as well as effective control, cleaning, and disinfection strategies for environmental moulds.

In meat processing plants, research is limited regarding the occupational exposure to *Aspergillus* and *Penicillium* and their mycotoxins. Moreover, to the best of the authors’ knowledge, there are no studies investigating the isolation and concentration of these moulds and mycotoxins on packaging materials used in meat processing plants. Additionally, their relationship to contamination of packaged products has not been explored. Furthermore, there is still little information on optimal strategies for environmental control, cleaning, and disinfection.

At meat markets, there is a lack of studies tracing the origin of *Aspergillus* and *Penicillium* and linking contamination to previous stages of the production chain or to contamination occurring at the market itself. Similarly, data on the isolation of moulds, including *Aspergillus* and *Penicillium*, from surfaces, air, and tools in meat markets, and their relationship with contamination of meat and meat products, are scarce.

This scoping review has some inherent limitations that should be acknowledged. Firstly, although careful and systematic procedures were followed, the screening and selection of studies were performed by a single author. Secondly, the number of databases searched is considered limited. In addition, the selection of databases may have affected which studies were included. PubMed was used as the primary source due to its high relevance and comprehensive coverage of literature within the scope of this review. While other databases such as Scopus, ScienceDirect, and Web of Science could have been included, they often overlap significantly with PubMed, and including them might have led to a high number of duplicate records. To complement this approach and capture a broader spectrum of studies, Google Scholar was also searched. This allowed for the identification of less-cited articles and publications in journals in the field of food microbiology and meat production with lower impact, which might not be indexed in PubMed. However, these studies may not have been subject to the same scientific rigour or indexing criteria as PubMed-indexed publications.

One final limitation of this study is that only species clearly identified within the *Aspergillus* and *Penicillium* genera were considered for inclusion, leading to the exclusion of species originally reported under outdated nomenclature that may currently belong to these genera. Consequently, some relevant data may not have been captured. A full taxonomic assessment of these species was not feasible within the scope of the study and would have required more extensive verification and standardisation. This approach was therefore adopted to focus on species with clearly identifiable, up-to-date names within *Aspergillus* and *Penicillium*, providing a clear and reproducible framework for species inclusion.

## 4. Conclusions

To the best of the authors’ knowledge, this is the first review addressing the occurrence of *Aspergillus* and *Penicillium* spp. across slaughterhouses, meat processing plants, and markets, highlighting their relevance for both food safety and occupational health. Although species-specific frequencies could not be compared due to methodological differences across studies, the evidence shows that these genera are present at all stages of meat and meat product production and across multiple matrices, including air, water, surfaces, equipment, raw materials, and final products. Their occurrence is likely underestimated in certain settings, particularly slaughterhouses, and several identified species are known mycotoxin producers, reinforcing their importance beyond spoilage. Fungal occurrence in the meat production chain is likely underestimated, as monitoring primarily targets bacteria. This is compounded by limited research, a lack of standardised protocols and guidelines for fungi, and low regulatory focus on mycotoxins, despite their role in spoilage and potential health risks. Fungal contamination in the meat sector is strongly influenced by environmental and technological factors, including temperature, humidity, hygiene practices, facility design, and handling conditions. The frequent detection of *Aspergillus* spp. in processing environments and among workers highlights the relevance of occupational exposure, which remains insufficiently explored.

Despite numerous reports of mould isolation, data on mycotoxin occurrence in meat matrices are limited and heterogeneous, underscoring the need for harmonised monitoring approaches. Overall, this review emphasises the integration of fungal surveillance into food safety management systems and the need for future research on mycotoxin production under real processing conditions, occupational exposure, and effective prevention and control strategies in the meat sector.

## Figures and Tables

**Table 1:** The most common mycotoxin-producing *Aspergillus* spp. previously detected on meat products [6,13,19,79].

*Aspergillus* Species	Mycotoxin
*A. flavus*	AFB_1_, Cyclopiazonic Acid (CA), 3-nitroproprionic acid
*A. niger*	OTA, Fumonisin B_2_
*A. nomius*	Aflatoxins B and G
*A. ochraceus*	OTA, Penicillic acid, Xanthomegnin, Viomellein, Vioxanthin
*A. parasiticus*	AFB_1_, AFG_1_
*A. tamarii*	AFG_1_, AFG_2_
*A. versicolor*	Sterigmatocystin
*A. westerdijkiae*	OTA

**Table 2:** *Aspergillus* species isolated from slaughterhouses, meat processing facilities, and markets (1990–2025).

Fungi Species	Location/Stage	Sort	Positive Sample(s)	Country
***A. flavus***	Slaughterhouse	Camel	Liver, lung, rumen, intestine	Egypt [37]
		Cattle	Air	Egypt [34]; Nigeria [56]; Serbia [64]
			Beef carcass	Egypt [34]; Nigeria [56]
			Floor	Egypt [34]; Serbia [64]
			Liver, lung, rumen, intestine, and head muscle	Egypt [37]
			Wall	Egypt [34]
			Water	Egypt [34]
			Slaughter ground scrapings	Nigeria [56]
		Sheep	Liver, lung, rumen, intestine, and head muscle	Egypt [37]
			Nasal swabs (butchers), meat swabs	Iraq [44]
		Poultry	Air	Austria [27]
		Non-defined	Air	Iraq [43]
			Houseflies	Iraq [45]
			Surfaces	Nigeria [55]
	Meat processing	Lamb/pork	Dry-cured meat production facility (outdoor air)	Norway [59]
		Mixed	Pork leg (14- and 20-month curing periods), pork shoulder, goat, and sheep	Portugal [13]
			Prime beef rump steak and milled black pepper in spring months, nutmeg in winter and spring months	Slovak Republic [65]
		Pork	Harbin dry sausages during fermentation	China [31]
			Sausage	Croatia [32]
		Non-defined	Sausage	Egypt [12]
	Market	Beef	Round muscle, neck muscles, masseter muscles, liver, and kidney	Saudi Arabia [38]
		Beef/poultry	Canned meat samples on DRBC; canned meat samples on Dichloran 18% Glycerol	Saudi Arabia [63]
		Bufallo	Round muscle, neck muscles, masseter muscles, liver, and kidney	Egypt [38]
		Mixed	Barčianska salami	Slovak Republic [65]
		Non-defined	Dried meat sample collected from five major markets	Nigeria [54]
			Dry Meat	Nigeria [57]
			Frozen meat samples on DRBC and PDA	Egypt [39]
			Sausage, beef burger, and minced meat (raw meat products), and hot dog (heat-treated meat product)	Egypt [40]
***A. fumigatus***	Slaughterhouse	Camel	Liver, lung, rumen, intestine, and head muscle	Egypt [37]
		Cattle	Air	Egypt [34]
			Beef carcass	Egypt [34]
			Floor	Egypt [34]
			Liver, lung, rumen, intestine, and head muscle	Egypt [37]
			Wall	Egypt [34]
			Water	Egypt [34]
		Sheep	Liver, lung, rumen, intestine, and head muscle	Egypt [37]
			Meat swabs	Iraq [44]
		Poultry	Air	Austria [27]; Italy [47]
			Lungs	Brazil [30]
		Non-defined	Houseflies	Iraq [45]
	Meat processing	Beef	Beefburger	Egypt [12]
		Lamb/pork	Dry-cured meat production facility (hams, environment, outdoor air)	Norway [59]
		Pork	Air	Italy [50]
		Non-defined	Dry-cured meat products	Norway [58]
	Market	Beef	Liver and kidney	Saudi Arabia [38]
		Bufallo	Liver	Egypt [38]
		Non-defined	Dried meat sample collected from five major markets	Nigeria [54]
			Dry Meat	Nigeria [57]
			Frozen meat samples on DRBC and PDA	Egypt [39]
			Luncheon meat samples from two companies	Egypt [35]
			Sausage, beef burger, and minced meat (raw meat products)	Egypt [40]
***A. niger***	Slaughterhouse	Camel	Liver, lung, rumen, intestine, and head muscle	Egypt [37]
		Cattle	Air	Egypt [34]; Nigeria [56]
			Beef carcass	Egypt [34]; Nigeria [56]
			Floor	Egypt [34]
			Liver, lung, rumen, intestine, and head muscle	Egypt [37]
			Slaughter ground scrapings	Nigeria [56]
			Wall	Egypt [34]
			Water	Egypt [34]
		Poultry	Air	Italy [47]
		Sheep	Nasal swabs (butchers), meat swabs	Iraq [44]
			Liver, lung, rumen, intestine, and head muscle	Egypt [37]
		Non-defined	Air	Iraq [43]
			Houseflies	Iraq [45]
			Surfaces	Nigeria [55]
	Meat processing	Lamb/pork	Dry-cured meat production facility (environment)	Norway [59]
		Mixed	Milled black pepper (summer and winter months) and nutmeg (spring months), emulsion Gombasek sausage (summer months)	Slovak Republic [65]
		Pork	Iberian ham	Spain [67]
		Non-defined	Sausage	Egypt [12]
	Market	Beef	Round muscle, neck muscles, masseter muscles, liver, and kidney	Saudi Arabia [38]
		Beef/poultry	Canned meat samples on DRBC and Dichloran 18% Glycerol	Saudi Arabia [63]
		Bufallo	Round muscle, neck muscles, masseter muscles, liver, and kidney	Egypt [38]
		Pork	Croatian traditional dry-cured meat products	Croatia [15]
		Non-defined	Dry Meat	Nigeria [57]
			Fresh and dried meat samples collected from five major markets	Nigeria [54]
			Frozen meat samples on DRBC and PDA	Egypt [39]
			Luncheon meat samples from two companies	Egypt [35]
			Sausage, beef burger, and minced meat (raw meat products), and luncheon (heat-treated meat product)	Egypt [40]
***A. nomius***	Meat processing	Mixed	Sheep (dry-cured ham)	Portugal [13]
***A. ochraceus***	Slaughterhouse	Camel	Rumen and head muscle	Egypt [37]
		Sheep	Rumen and intestine	Egypt [37]
		Non-defined	Houseflies	Iraq [45]
	Meat processing	Pork or mixed	Sausage	Argentina [24,25]; Croatia [32]
		Non-defined	Sausage	Egypt [12]
	Market	Beef	Neck muscle and kidney	Saudi Arabia [38]
		Beef/poultry	Canned meat samples on DRBC and Dichloran 18% Glycerol	Saudi Arabia [63]
		Bufallo	Neck muscle and kidney	Egypt [38]
		Pork	Croatian traditional dry-cured meat products	Croatia [15]
		Non-defined	Dry Meat	Nigeria [57]
			Sausage and beef burger (raw meat products), and luncheon, hot dog, and canned meat (heat-treated meat products)	Egypt [40]
***A. parasiticus***	Slaughterhouse	Non-defined	Houseflies	Iraq [45]
	Meat processing	Mixed	Goat (dry-cured ham)	Portugal [13]
			Sheep (dry-cured ham)	Portugal [13]
		Pork or mixed	Air samples of processing rooms	Argentina [24]
			Sausage	Argentina [24]; Croatia [32]
		Non-defined	Sausage	Egypt [12]
	Market	Non-defined	Beef burger and minced meat (raw meat products)	Egypt [40]
***A. sydowii***	Slaughterhouse	Cattle	Air, water, floor, wall, beef inner surface of shoulder, and beef inner surface of thigh	Egypt [34]
	Meat processing	Lamb/pork	Dry-cured meat production facility (environment)	Norway [59]
		Pork	Iberian ham	Spain [67]
			Sausage	Croatia [10]
	Market	Non-defined	Dried meat sample collected from five major markets	Nigeria [54]
			Luncheon meat samples from one company	Egypt [35]
***A. tamarii***	Meat processing	Pork	Harbin dry sausages during fermentation	China [31]
	Market	Non-defined	Dry Meat	Nigeria [57]
			Fresh and dried meat samples were collected from five major markets	Nigeria [54]
***A. terreus***	Slaughterhouse	Camel	Liver, rumen, intestine, lung, and head muscle	Egypt [37]
		Cattle	Air	Egypt [34]; Serbia [64]
			Beef carcass	Egypt [34]
			Floor	Egypt [34]; Serbia [64]
			Liver, rumen, intestine, and head muscle	Egypt [37]
			Wall	Egypt [34]; Serbia [64]
		Sheep	Rumen, intestine, and head muscle	Egypt [37]
	Market	Beef	Neck muscles, liver, and kidney	Saudi Arabia [38]
		Bufallo	Liver	Egypt [38]
		Non-defined	Fresh and dried meat samples collected from five major markets	Nigeria [54]
			Luncheon meat samples from two companies	Egypt [35]
			Sausage (raw meat product)	Egypt [40]
***A. versicolor***	Slaughterhouse	Camel	Liver, rumen, and head muscle	Egypt [37]
		Cattle	Air, water, floor, wall, beef carcass outer surface of shoulder, and beef inner surface of thigh	Egypt [34]
			Intestine	Egypt [37]
		Sheep	Lung, rumen, and intestine	Egypt [37]
	Meat processing	Lamb/pork	Dry-cured meat production facility (environment)	Norway [59]
		Pork	Dry-cured pork neck and dry-cured ham	Slovenia [66]
			Iberian ham	Spain [67]
			Sausage	Croatia [10]; Italy [11,50]
		Non-defined	Air	Brazil [29]
	Market	Pork	Croatian traditional dry-cured meat products	Croatia [15]
***A. westerdijkiae***	Meat processing	Pork	Pork leg (20 months curating period)	Portugal [13]
			Sausage	Italy [50]
		Non-defined	Air, sausage	Brazil [29]

DRBC: Dichloran Rose Bengal Chloramphenicol agar; PDA: Potato Dextrose Agar.

**Table 3:** The most common mycotoxin-producing *Penicillium* spp. previously detected on meat products [6,19,79].

*Penicillium* Species	Mycotoxin
*P. aurantiogriseum*	Penicillic acid (PA), verrucosidin, terrestric acid, nephrotoxic glycopeptides
*P. brevicompactum*	Botryodiploidin
*P. chrysogenum*	Secalonic acid, PR toxin, roquefortine C
*P. citrinum*	Citrinin
*P.commune*	Cyclopiazonic acid (CA)
*P. crustosum*	Terrestric acid, penitrems, roquefortine C
*P. cyclopium*	PA, xanthomegnins
*P. expansum*	Patulin, citrinin, chaetoglobosins, communesins, roquefortine C
*P. glabrum*	Citromycetin
*P. griseofulvum*	Patulin, griseofulvin, roquefortine C, CA
*P. nordicum*	OTA, viridic acid (VA)
*P. oxalicum*	Secalonic acids, roquefortine C
*P. palitans*	CA
*P. polonicum*	OTA, CA
*P. roquefortii*	PR toxin, roquefortine C
*P. rugulosum*	Rugulosin
*P. variabile*	Rugulosin
*P. verrucosum*	OTA, citrinin
*P. viridicatum*	PA, xanthoemegnins, VA

**Table 4:** *Penicillium* species isolated from slaughterhouses, meat processing facilities, and markets (1990–2025).

Fungi Species	Location/Stage	Sort	Positive Sample(s)	Country
***P. aurantiogriseum***	Slaughterhouse	Cattle	Air, water, wall, beef carcass	Egypt [34]
		Non-defined	Houseflies	Iraq [45]
	Meat processing	Pork	Air of dry-cured ham manufacturing plants	Italy [46]
			Iberian ham	Spain [67]
	Market	Beef/poultry	Canned meat samples on DRBC	Saudi Arabia [63]
		Non-defined	Luncheon meat samples from two companies	Egypt [35]
***P. brevicompactum***	Slaughterhouse	Cattle	Air, floor, and wall	Serbia [64]
	Meat processing	Beef/pork	Fermented sausage from two companies (spring and autumn)	Denmark [33]
		Lamb/pork	Dry-cured meat production facility (hams, Fenalår, environment, air)	Norway [59]
		Mixed	Pork leg (20-month curing period), pork shoulder, goat, and sheep	Portugal [13]
		Pork or mixed	Air	Argentina [24]; Italy [46]
			Dry-cured pork neck and dry-cured ham	Slovenia [66]
			Two liver pâté from plants (spring and autumn)	Denmark [33]
			Sausage	Argentina [24,25]; Italy [50]
		Non-defined	Air	Brazil [29]
			Dry-cured meat product	Norway [58]
	Market	Pork	All types of Speck (crust, fat, and meat)	Austria/Italy [26]
			Croatian traditional dry-cured meat products	Croatia [15]
			Fat and meat of the Speck from farmers, meat from butcheries, and fat from industries	Italy [26]
			Meat of the Speck (farmers) and fat and meat of the Speck (industries)	Austria [26]
***P. chrysogenum***	Slaughterhouse	Cattle	Air	Egypt [34]; Serbia [64]
			Beef carcass	Egypt [34]
			Floor	Egypt [34]; Serbia [64]
			Wall	Egypt [34]; Serbia [64]
			Water	Egypt [34]
	Meat processing	Beef/pork/small ruminants	Fermented sausage plant (autumn)	Denmark [33]
			Pork leg (14-month curing period), goat, and sheep	Portugal [13]
		Beef	Beefburger	Egypt [12]
		Lamb/pork	Dry-cured meat production facility (hams, Fenalår, environment, air)	Norway [59]
		Pork or mixed	Air	Argentina [24]; Italy [46]
			Dry-cured pork neck	Slovenia [66]
			Ham muscle	Italy [46]
			Iberian ham	Spain [67]
			Sausage	Argentina [24]; Croatia [32]; Italy [11,50]
		Non-defined	Dry-cured meat products	Norway [58]
			Sausage	Brazil [29]
	Market	Beef/poultry	Canned meat samples on DRBC and Dichloran 18% Glycerol	Saudi Arabia [63]
		Pork	All types of Speck (crust, fat, and meat)	Austria/Italy [26]
			Croatian traditional dry-cured meat products	Croatia [15]
			Fat and meat of the Speck from farmers and industries	Austria [26]
			Fat and meat of the Speck (industries)	Italy [26]
		Non-defined	Dried meat sample collected from five major markets	Nigeria [54]
			Luncheon meat samples from two companies	Egypt [35]
***P. citrinum***	Meat processing	Pork	Air	Italy [46]
			Ham muscle	Italy [46]
			Ham portion	Italy [46]
			Harbin dry sausages during fermentation	China [31]
			Pork leg (20 months curing period)	Portugal [13]
			Sausage	Croatia [32]
		Non-defined	Air, sausage casing	Brazil [29]
			Dry-cured meat product	Norway [58]
	Market	Beef/poultry	Canned meat samples on DRBC and Dichloran 18% Glycerol	Saudi Arabia [63]
		Non-defined	Frozen meat samples on DRBC and PDA	Egypt [39]
***P. commune***	Meat processing	Beef	Sausage	Japan [51]
		Beef/pork	Two fermented sausage plants (spring and autumn)	Denmark [33]
		Lamb/pork	Dry-cured meat production facility (hams, environment, air)	Norway [59]
		Mixed	Pork leg (14- and 20-month curing periods), pork shoulder, goat, and sheep	Portugal [13]
		Pork or mixed	Iberian ham	Spain [67]
			Sausage	Argentina [24]; Croatia [32]; Italy [11]
		Non-defined	Dry-cured meat product	Norway [58]
	Market	Pork	All types of Speck (crust, fat, and meat)	Austria/Italy [26]
			Croatian traditional dry-cured meat products	Croatia [15]
			Fat and meat of the Speck from butcheries and farmers	Austria [26]
			Fat and meat of the Speck from farmers and butcheries	Italy [26]
		Non-defined	Visibly mouldy traditional Greek sausages	Greece [41]
***P. crustosum***	Meat processing	Lamb/pork	Dry-cured meat production facility (hams, Fenalår, environment, air)	Norway [59]
		Mixed	Pork leg (14- and 20-month curing periods), pork shoulder, goat, and sheep	Portugal [13]
		Pork	Air	Italy [46]
		Non-defined	Dry-cured meat products	Norway [58]
			Sausage	Brazil [29]
	Market	Non-defined	Frozen meat samples on DRBC and PDA	Egypt [39]
***P. cyclopium***	Meat processing	Beef/pork	Fermented sausage plant in autumn	Denmark [33]
		Beef	Beefburger	Egypt [12]
		Mixed	Goat	Portugal [13]
		Non-defined	Kubeba, fresh meat	Egypt [12]
***P. glabrum***	Meat processing	Beef/pork	Fermented sausage plant in autumn	Denmark [33]
		Lamb/pork	Dry-cured meat production facility (hams, environment, air)	Norway [59]
		Pork or mixed	Air	Argentina [24]; Italy [46]
			Two liver pâté plants (spring and autumn)	Denmark [33]
		Non-defined	Air, sausage	Brazil [29]
	Market	Pork	Fat of the Speck (industries) and meat of the Speck (farmers)	Austria [26]
			Fat and meat samples of Speck originating from farmers, butcheries, and industrial producers	Italy [26]
			All types of Speck (crust, fat, and meat)	Austria/Italy [26]
***P. griseofulvum***	Meat processing	Pork	Air	Italy [46]
			Sausage	Italy [11,50]
		Non-defined	Air, sausage	Brazil [29]
	Market	Beef/poultry	Canned meat samples on DRBC agar and on Dichloran 18% Glycerol medium	Saudi Arabia [63]
		Non-defined	Frozen meat samples on DRBC and PDA media	Egypt [39]
***P. expansum***	Slaughterhouse	Poultry	Air	Italy [47]
	Meat processing	Lamb	Dry-cured meat production facility (Fenalår)	Norway [59]
		Pork	Air	Italy [46]
			Iberian ham	Spain [67]
			Sausage	Croatia [32]; Italy [11];
		Non-defined	Sausage	Egypt [12]
			Dry-cured meat products	Norway [58]
	Market	Non-defined	Visibly mouldy traditional Greek sausages	Greece [41]
***P. italicum***	Meat processing	Lamb/pork	Dry-cured meat production facility (air)	Norway [59]
		Non-defined	Sausage	Brazil [29]; Egypt [12]
	Market	Non-defined	Visibly mouldy traditional Greek sausages	Greece [41]
***P. nordicum***	Meat processing	Pork or mixed	Air	Argentina [24]; Italy [46]
			Ham muscle	Italy [46]
			Pork leg (20-month curing period) and pork shoulder	Portugal [13]
			Salami, dry-cured pork neck and dry-cured ham	Slovenia [66]
			Sausage	Argentina [24]; Italy [11,50]; Slovenia [66]
***P. palitans***	Meat processing	Beef/pork	Fermented sausage plant in autumn	Denmark [33]
		Lamb/pork	Dry-cured meat production facility (Hams, Fenalår, Environment, Air)	Norway [59]
		Non-defined	Dry-cured meat products	Norway [58]
***P. polonicum***	Slaughterhouse	Poultry	Air	Italy [47]
	Meat processing	Pork	Pork leg (20 months curing period)	Portugal [13]
			Salami and dry-cured ham	Slovenia [66]
			Sausage	Croatia [10]
		Non-defined	Sausage	Brazil [29]
	Market	Pork	Croatian traditional dry-cured meat products	Croatia [15]
***P. roqueforti***	Meat processing	Beef or pork	Fermented sausage plant (spring)	Denmark [33]
		Lamb	Dry-cured meat production facility (Fenalår)	Norway [59]
		Pork	Air	Italy [46]
			Ham muscle	Italy [46]
		Non-defined	Dry-cured meat products	Norway [58]
			Sausage	Brazil [29]
	Market	Pork	Croatian traditional dry-cured meat products	Croatia [15]
***P. rugulosum***	Meat processing	Pork	Iberian ham	Spain [67]
***P. variabile***	Slaughterhouse	Cattle	The outer surfaces of beef carcasses, including the shoulder and thigh	Egypt [34]
	Meat processing	Non-defined	Sausage	Egypt [12]
	Market	Non-defined	Luncheon meat samples from a specific company	Egypt [35]
***P. verrucosum***	Slaughterhouse	Non-defined	Houseflies	Iraq [45]
	Meat processing	Pork	Sausage	Croatia [32]; Italy [11]
	Market	Pork	All types of Speck (crust, fat, and meat)	Austria/Italy [26]
			Croatian traditional dry-cured meat products	Croatia [15]
			Fat and meat of the Speck from butcheries, farmers, and industries	Italy [26]
			Fat of the Speck (industries)	Austria [26]; Italy [26]
		Non-defined	Visibly mouldy traditional Greek sausages	Greece [41]
***P. viridicatum***	Meat processing	Mixed	Pork leg (20 months curing period)	Portugal [13]
		Pork or mixed	Iberian ham	Spain [67]
			Sausage	Argentina [24]; Italy [11]
		Non-defined	Air	Brazil [29]
	Market	Non-defined	Luncheon meat samples from two companies	Egypt [35]
			Visibly mouldy traditional Greek sausages	Greece [41]

DRBC: Dichloran Rose Bengal Chloramphenicol agar; PDA: Potato Dextrose Agar.

## Data Availability

The original contributions presented in this study are included in the article/Supplementary Materials. Further inquiries can be directed to the corresponding author.

## Supplementary Materials

The following supporting information can be downloaded at: https://www.mdpi.com/article/10.3390/foods15040630/s1, Table S1: Detection of mycotoxins in offal, meat, and meat products from slaughterhouses, meat manufacturers, and meat markets across 10 studies (1990–2025); Table S2: Overview of fungal contamination, focused on *Aspergillus* spp. and *Penicillium* spp., reported in slaughterhouses across 17 studies (1990–2025); Table S3: Overview of fungal contamination, focused on *Aspergillus* spp. and *Penicillium* spp., reported in meat manufacturers across 24 studies (1990–2025); Table S4: Overview of fungal contamination, focused on *Aspergillus* spp. and *Penicillium* spp., reported in retail meat markets across 14 studies (1990–2025); Table S5: Overview on other isolated *Aspergillus* spp. from samples in slaughterhouses, meat manufacturers, and retail meat markets; Table S6: Overview on other isolated *Penicillium* spp. from samples in slaughterhouses, meat manufacturers, and retail meat markets.
