# Supplementary material for: Moulds and Mycotoxins in the Meat Production Chain from Slaughterhouse to Market: A Scoping Review on Aspergillus and Penicillium Isolation
Source: Foods. 2026 Feb 9;15(4):630. doi: 10.3390/foods15040630 (PMC12939195; doi:10.3390/foods15040630)
Supplement: Supplementary file 1 [file foods-15-00630-s001.zip › foods-4143467-supplementary.pdf]

Table S1. Detection of mycotoxins in offal, meat, and meat products from slaughterhouses, meat manufacturers, and meat markets across 10 studies (1990-2025).

Local	Country	Species	Type of sample	N	Types of mycotoxins	Positive samples (n)	Positive samples (%)	Concentration Range (μg/kg)	Concentration Mean (μg/kg)	LOD (μg/kg)	Analytical Method	References
**Slaughterhouse**	Mozambique	Poultry	Industrial gizzards	50	AFB1	2	4.0	0.81–1.34	1.07±0.37	NR	ELISA	[9]
			Industrial liver	70	AFB1	19	27.1	0.61–2.48	1.35 ±0.58	NR	ELISA	
			Local sector producer gizzards	30	AFB1	9	30.0	0.68–2.12	1.04 ±0.44	NR	ELISA	
			Local sector producer liver	30	AFB1	20	66.7	0.57–3.80	1.73 ±1.09	NR	ELISA	
**Meat processing plants / Meat producers**	Croatia	Pork	Slavonski kulen (6 months of production)	9	OTA	2	22.2	1.91-2.43	2.17 ± 0.37	0.9	ELISA	[10]
			Slavonski kulen (9 months of production)	9	AFB1	3	33.3	2.34-2.78 (external layer)	2.56 ±0.31	1.2	ELISA	
								1.62-1.62 (internal layer)	1.62 ± 0	1.2	ELISA	
			Slavonski kulen (9 months of production)	9	OTA	5	55.6	2.06-3.91 (external layer)	3.23 ± 1.02	0.9	ELISA	
								1.37-2.72 (internal layer)	2.05 ± 0.95	0.9	ELISA	
			Slavonski kulen (12 months of production)	9	AFB1	7	77.8	10.14-14.46 (external layer)	11.79 ± 2.34	1.2	ELISA	
								2.72-3.97 (internal layer)	3.28 ±0.64	1.2	ELISA	
								1.84-2.19 (central layer)	2.02 ± 0.25	1.2	ELISA	
			Slavonski kulen (12 months of production)	9	OTA	9	100.0	13.42-19.84 (external layer)	16.13 ± 3.32	0.9	ELISA	
								2.81-4.23 (internal layer)	3.72 ± 0.79	0.9	ELISA	
								1.97-2.52 (central layer)	2.23 ± 0.28	0.9	ELISA	

Table S1. *(continued)*

Local	Country	Species	Type of sample	N	Types of mycotoxins	Positive samples (n)	Positive samples (%)	Concentration Range (μg/kg)	Concentration Mean (μg/kg)	LOD (μg/kg)	Analytical Method	References
**Meat processing plants / Meat producers**	Italy	Pork	Bergamasco sausage (Artisanal)	12	OTA	5	41.7	5–12	5.8 ± 3.0	0.1	ELISA/HPLC	[11]
			Bergamasco sausage (Industrial)	6	OTA	3	50.0	5–12	8.0 ± 1.5	0.1	ELISA/HPLC	
			Brianza sausage (Artisanal)	10	OTA	5	50.0	3–9	6.0 ± 1.0	0.1	ELISA/HPLC	
			Brianza sausage (Industrial)	10	OTA	6	60.0	3–9	6.0 ± 0.5	0.1	ELISA/HPLC	
			Cremonese sausage (Artisanal)	12	OTA	5	41.7	3–10	5.8 ±0.5	0.1	ELISA/HPLC	
			Cremonese sausage (Industrial)	6	OTA	4	66.7	3–10	5.5 ± 0.5	0.1	ELISA/HPLC	
			Mantovano sausage (Artisanal)	12	OTA	4	33.3	4–15	7.5 ± 2.5	0.1	ELISA/HPLC	
			Mantovano sausage (Industrial)	6	OTA	3	50.0	4–15	6.0 ±0.5	0.1	ELISA/HPLC	
			Milano sausage (Artisanal)	20	OTA	5	25.0	3–12	4.9 ±3.5	0.1	ELISA/HPLC	
			Milano sausage (Industrial)	10	OTA	6	60.0	3–12	4.5 ± 2.5	0.1	ELISA/HPLC	
			Napoli sausage (Artisanal)	7	OTA	3	42.9	4–13	7.0 ± 1.0	0.1	ELISA/HPLC	
			Napoli sausage (Industrial)	6	OTA	4	66.7	4–13	7.5 ± 0.5	0.1	ELISA/HPLC	
			Ungherese sausage (Artisanal)	7	OTA	3	42.9	3–18	6.0 ± 2.5	0.1	ELISA/HPLC	
			Ungherese sausage (Industrial)	6	OTA	4	66.7	3–18	5.0 ± 1.5	0.1	ELISA/HPLC	
			Varzi sausage (Artisanal)	20	OTA	7	35.0	5–10	6.0 ± 1.5	0.1	ELISA/HPLC	
			Varzi sausage (Industrial)	10	OTA	5	50.0	5–10	5.6 ± 3.0	0.1	ELISA/HPLC	

Table S1. *(continued)*

Local	Country	Species	Type of sample	N	Types of mycotoxins	Positive samples (n)	Positive samples (%)	Concentration Range (µg/kg)	Concentration Mean (µg/kg)	LOD (µg/kg)	Analytical Method	References
**Meat processing plants / Meat producers**	Egypt	Mixed	Beefburger	25	AFB1	5	20.0	NR	8	NR	TLC	[12]
			Black pepper	15	AFB1	4	26.7	NR	35	NR	TLC	
			Coriander	15	AFB1, AFG1	2	13.3	NR	8 (AFB1)	NR	TLC	
									2 (AFG1)	NR	TLC	
			Hot-dog	25	AFB1, AFB2	1	4.0	NR	5 (AFB1)	NR	TLC	
									2 (AFB2)	NR	TLC	
			Kubeba	25	AFB1, AFB2	1	4.0	NR	150 (AFB1)	NR	TLC	
									25 (AFB2)	NR	TLC	
			Luncheon meat	25	AFB1, AFB2	1	4.0	NR	4 (AFB1)	NR	TLC	
									2 (AFB2)	NR	TLC	
			Sausage	25	AFB1, AFB2	1	4.0	NR	7 (AFB1)	NR	TLC	
									3 (AFB2)	NR	TLC	
			Turmeric	15	AFB1, AFG1	2	13.3	NR	12 (AFB1)	NR	TLC	
									8 (AFG1)	NR	TLC	
			White pepper	15	AFB1	4	26.7	NR	22	NR	TLC	
	Portugal	Pork	Fresh meat	15	OTA	6	40.0	<LOQ (0.9)	<LOQ	0.3	HPLC-FLD	[13]
			Leg (curing period 14 months)	9	OTA	1	11.0	<LOQ (0.9)	<LOQ	0.3	HPLC-FLD	
			Leg (curing period 20-25 months)	47	OTA	20	43.0	<LOQ (0.9) – 99.1	14.9±27.9	0.3	HPLC-FLD	
			Shoulder	40	OTA	7	48.0	<LOQ (0.9)– 14.7	6.6±6.2	0.3	HPLC-FLD	
	Spain	Pork	Iberian ham (superficial portion)	20	OTA	10	50.0	2.0-160.9	NR	1	HPLC–MS	[14]
			Iberian ham (deep portion)	20	OTA	3	15.0	2.0 -28.4	NR	1	HPLC–MS	

Table S1. *(continued)*

Local	Country	Species	Type of sample	N	Types of mycotoxins	Positive samples (n)	Positive samples (%)	Concentration Range (µg/kg)	Concentration Mean (µg/kg)	LOD (µg/kg)	Analytical Method	References
**Meat market**	Croatia	Pork	Dalmatian prosciutto	32	AFB1	4	12.5	1.51-1.91	1.73±0.17	0.95	ELISA	[15]
			Dalmatian prosciutto	32	OTA	5	15.6	2.16-5.39	3.56±1.32	0.91	ELISA	
			Istrian domestic sausage	41	AFB1	2	4.9	1.53-1.71	1.62±0.13	1.06	ELISA	
			Istrian domestic sausage	41	OTA	3	7.3	2.74-3.12	2.97±0.20	1.15	ELISA	
			Istrian prosciutto	35	AFB1	5	14.2	1.56-1.92	1.7±0.16	0.95	ELISA	
			Istrian prosciutto	35	OTA	10	28.6	2.74-6.86	5.04±1.5	0.91	ELISA	
			Kulenova seka	25	OTA	2	8.0	2.85-3.42	3.14±0.4	1.15	ELISA	
			Slavonian domestic sausage	27	AFB1	1	3.7	1.83-1.83	1.83±0.00	1.06	ELISA	
			Slavonian domestic sausage	27	OTA	2	7.4	3.64-4.14	3.89±0.35	1.15	ELISA	
			Dry fermented sausages (industrial)	56	OTA	18	32.1	1.36 - 7.12	3.02 ± 2.45	0.84	ELISA	[16]
			Dry fermented sausage (homemade)	77	OTA	11	14.3	1.36 - 6.26	3.54 ± 1.70	0.84	ELISA	
			Dry-cured ham	54	OTA	12	22.1	1.56 - 9.95	3.16 ± 2.42	0.32	ELISA	
	Cyprus	Sheep and goat	Samerella samples (values of results that above LOQ)	30	AF	14	47.0	5.3–12.5	10.3	0.05	ELISA	[17]
					AFB1	9	30.0	0.10–0.12	0.11	0.1	ELISA	
	Italy	Pork	Calabrian salamis	50	OTA	13	26.0	0.07-0.62	0.18±0.14	0.05	HPLC-FLD	[18]
			Piedmontese salamis	52	OTA	4	7.7	0.13-5.66	1.86±2.61	0.05	HPLC-FLD	
			Salamis (all types)	172	OTA	22	12.8	0.07-5.66	0.51±0.32	0.05	HPLC-FLD	
			Sicilian salamis	54	OTA	5	9.3	0.07-1.3	0.29±0.42	0.05	HPLC-FLD	

Table S2. Overview of fungal contamination, focused on *Aspergillus* spp. and *Penicillium* spp., reported in slaughterhouses across 17 studies (1990-2025).

No.	Country	Highlights	Reference
1	Austria	A case study in a poultry slaughterhouse in Styria, Austria, measured airborne microorganisms at the 'moving rail' and 'gall bladder separation' sites. At one location, among other airborne fungal species, *Penicillium* spp. accounted for 11%, *Aspergillus* spp. for 10%, *A. fumigatus* for 2%, and *A. flavus* for less than 1%. At another location, *Penicillium* spp. represented 10%, *A. fumigatus* 8%, and *Aspergillus* spp. 2%. Airborne fungal spore loads were low compared to the loads of mesophilic bacteria.	[27]
2	Brazil	Moulds, particularly *Aspergillus* spp., are a significant cause of lung disease in poultry, with infection occurring via inhalation of airborne conidia. In this study, lungs from 76 slaughtered broilers with airsacculitis were analysed, and *Aspergillus fumigatus* sensu stricto was identified as the primary etiological agent using mycological, histopathological, and molecular methods.	[30]
3	Egypt	A total of 34 fungal genera were isolated from beef carcasses and their surroundings. *Aspergillus* was frequently found on carcasses and in the environment, with *A. flavus* and *A. niger* showing moderate incidence on carcasses but high incidence in surrounding air, water, floors, and walls. Other *Aspergillus* species, as well as *Cladosporium* and *Penicillium*, were common in the environment but rarely recovered from carcasses, suggesting that the surroundings are an important source of mould contamination.	[34]
4	Egypt	This study assessed total mould counts and the occurrence of different mould genera in raw edible offal from cattle, camels, and sheep in Zagazig, Egypt. Cattle offal showed the highest mould counts, followed by camel and sheep, with intestines being the most contaminated organ across all species. *Aspergillus* and *Penicillium* were the predominant genera, with *Aspergillus niger* being the most frequent species, highlighting the need for interventions, such as chemical or natural additives, to reduce contamination.	[37]

Table S2. *(continued)*

No.	Country	Highlights	Reference
5	India	Fungal species identified at the slaughterhouse included *Alternaria*, *Aspergillus*, *Penicillium*, and *Candida.*	[42]
6	Iraq	This study compared airborne fungi inside and around a slaughterhouse. Five fungal species were identified, including *Aspergillus flavus*, *Aspergillus niger*, *Penicillium notatum*, and other *Penicillium* species. *Penicillium notatum* showed the highest frequency (26.25%) both inside and outside the slaughterhouse, while *Aspergillus niger* was present only in the outside air.	[43]
7	Iraq	The study investigated zoonotic bacteria and fungi in sheep, butchers, and slaughterhouse equipment in Baghdad. Among the fungal isolates from skin scrapings, *Aspergillus fumigatus*, *Aspergillus flavus*, and *Aspergillus niger* were detected, alongside other fungi. These findings indicate that sheep and slaughterhouse environments can serve as reservoirs for *Aspergillus* species.	[44]
8	Iraq	This study investigated the role of house flies (*Musca domestica*) from slaughterhouses in Al-Diwaniyah, Iraq, as carriers of bacterial and fungal pathogens. From 120 collected flies, 154 fungal isolates belonging to 16 species were obtained from their external surfaces, with *Aspergillus niger* being the most frequent fungal species. *Penicillium* species were also detected among the fungal isolates, indicating that house flies can act as vectors for these moulds and potentially contribute to the spread of fungal pathogens in human and animal environments.	[45]
9	Italy	This study characterised airborne bacteria, fungi, and endotoxin concentrations in two Italian poultry slaughterhouses. Fungal contamination was lower than bacterial levels but included species from the *Cladosporium*, *Penicillium*, and *Aspergillus* genera.	[47]
10	Korea	This study investigated fungal contamination in four pig slaughterhouses in Korea. *Aspergillus niger* and the *Penicillium* species *P. commune* and *P. oxalicum* were among the most frequently detected fungi.	[53]

Table S2. *(continued)*

No.	Country	Highlights	Reference
11	Nigeria	This study found notable fungal contamination on slaughter slab surfaces in the Ado-Ekiti central abattoir, with *Aspergillus flavus* being the most prevalent species (~40%), followed by *Penicillium* spp. (28%). These findings indicate a significant presence of mould on contact surfaces, reflecting poor hygiene and a high risk of meat contamination.	[55]
12	Nigeria	This study found a high prevalence of fungi in the Yola abattoir environment, with *Aspergillus flavus* (17.6%) and *Aspergillus niger* (15.1%) being the most common species, followed by *Penicillium* spp. (14.5%) and *Trichophyton rubrum* (14.5%).	[56]
13	Pakistan	This 12-month survey of four slaughterhouses in Lahore reported high levels of airborne fungi, with *Penicillium* spp. (29.3%) and *Aspergillus* spp. (18.0%) among the most frequently isolated genera.	[60]
14	Portugal	This study assessed fungal contamination in poultry, swine/bovine and large-animal slaughterhouses. *Penicillium* spp. were highly prevalent in the large-animal facility (80.8%). *Aspergillus fumigatus* complex (3.13%), *A. ochraceus* complex (5.5%), and *A. terreus* complex (5.5%) were also isolated in the study. The qPCR detected *Aspergillus fumigatus* complex DNA in six sampling sites where conventional culture methods failed to identify it.	[61]
15	Saudi Arabia	This study assessed indoor air quality and surface contamination in the Dammam slaughterhouse. Among the fungal contaminants, *Aspergillus* species were detected, appearing in air samples and on utensils. *Penicillium* spp. were isolated in a laboratory's air samples alongside other yeasts and dermatophytes.	[62]
16	Serbia	The study examined fungal contamination in two slaughterhouses by collecting samples from air, water, surfaces, and equipment. *Aspergillus* and *Penicillium* were the predominant moulds detected in air, water, on surfaces, and on equipment in both slaughterhouses.	[64]
17	Sweden	In the hanging departments of four poultry slaughterhouses, airborne fungal levels were lower than bacterial levels; however, the fungal flora included *Penicillium* and *Aspergillus*, among other moulds. Despite low concentrations, these fungi contribute to occupational exposure in workers handling poultry.	[69]

Table S3. Overview of fungal contamination, focused on *Aspergillus* spp. and *Penicillium* spp., reported in meat manufacturers across 24 studies (1990-2025).

No.	Country	Highlights	Reference
1	Argentina	This study examined fungal diversity in fermented sausage plants in southern Buenos Aires, Argentina. Among the isolates, *Penicillium* species, especially *P. nalgiovense*, *P. nordicum*, *P. solitum*, and *P. chrysogenum*, were most common. Fungal composition varied by season and humidity, with *Aspergillus* spp. more frequent in summer.	[24]
2	Argentina	This study analysed the surface fungi of dry fermented sausages from Colonia Caroya, Argentina, produced without fungal starters. Across seasons, *Penicillium nalgiovense* biotype 4 dominated nearly all samples, giving sausages a grey-white colour and posing no mycotoxin risk. In contrast, *Aspergillus ochraceus* appeared in 80–90% of summer sausages—linked to high temperatures and poor ripening control, causing yellow discolouration and economic losses.	[25]
3	Brazil	This study analysed fungal contamination during the processing of seasoned and unseasoned raw sheep hams. *Aspergillus* species became dominant as ripening progressed, spreading through airborne spores between products. At the end of ripening, xerophilic *Aspergillus* prevailed, and a potentially ochratoxigenic, *A. circumdati*, appeared on unseasoned hams.	[28]
4	Brazil	This study evaluated fungal contamination in raw materials, sausage surfaces, and air from two Brazilian dry sausage plants. Raw materials showed low contamination, while sausage surfaces harboured diverse fungi, mainly *Aspergillus* and *Penicillium*. Airborne fungal counts were high, especially in a specific industry, where *Aspergillus westerdijkiae*, an ochratoxin-producing species, was detected in both air and sausages.	[29]
5	China	The study examined the succession of fungal communities and the dynamics of volatile compounds in Harbin dry sausage during a 12-day fermentation period. *Aspergillus pseudoglaucus* was identified as the dominant species, with *A. caesiellus*, *A. gracilis*, and other fungi also present. Correlation analysis showed that several core fungi, especially *A. pseudoglaucus*, *A. gracilis*, *Trichosporon caseorum*, *Debaryomyces hansenii*, and *T. asahii*, were positively associated with these key flavour compounds.	[31]
6	Croatia	The study found that the surface moulds on Istrian ham mainly originated from the curing environment. The dominant fungi were *Aspergillus*, *Penicillium*, and *Eurotium*. None of the isolated *Aspergillus* or *Penicillium* strains produced mycotoxins, indicating no health risk from these moulds.	[32]
7	Croatia	This study examined mycotoxin contamination and fungal species on *slavonski kulen* during 12 months of production. Higher levels of aflatoxin B1 and ochratoxin A were found on the sausage surface than in the interior. The main fungi identified were *Penicillium* (five species) and *Aspergillus* (two species).	[10]
8	Denmark	This study found diverse mould contamination in meat plants, with *Penicillium* as the main genus in all facilities. Key species included *P. brevicompactum*, *P. solitum*, and *P. palitans*, some of which produce mycotoxins such as mycophenolic and cyclopiazonic acids, posing potential risks for contamination of meat products.	[33]
9	Egypt	A survey in Cairo, Egypt, found that processed meat products and spices were contaminated with aflatoxins, while fresh and canned meats were not. *Aspergillus flavus* and *A. parasiticus* were the main aflatoxin-producing moulds.	[12]
10	Italy	This study examined ochratoxin A–producing moulds on northern Italian sausage casings. Among the isolated strains, the most frequent were *Penicillium nalgiovense*, *P. oxalicum*, *Eurotium amstelodami*, and other *Penicillium* species. *Aspergillus ochraceus* was rare. About 45% of samples had OTA on the casings, but brushing and washing reduced it below detection levels, and OTA was absent inside the meat, indicating no significant health risk for consumers.	[11]

Table S3. *(continued)*

No.	Country	Highlights	Reference
11	Italy	A year-long study of seven Italian ham plants examined fungi in air and on hams, focusing on potential mycotoxin producers. *Aspergillus* and *Penicillium* were found in all plants, with *P. nalgiovense* dominating (~60%) and *P. nordicum* present at 10–26%. About 50% of *P. nordicum* isolates could produce ochratoxin A in vitro. Contamination was higher in warmer ripening rooms, and a correlation existed between air and ham surface contamination. Although *P. nordicum* is present, it is not the dominant species.	[46]
12	Italy	This study investigated the antimicrobial activity of gaseous ozone in a meat production plant. Ozone up to 20 ppm had no inhibitory effect on moulds (*Aspergillus niger*, *Penicillium roqueforti*, *Mucor racemosus*). Results highlight that ozone's antifungal efficacy depends on environmental and technical conditions in real food-processing settings.	[48]
13	Italy	This study investigated the fungal community on salami during the seasoning process at a Calabria plant. One species was identified as *Penicillium nalgiovense*, and the other was related to, but distinct from, *Penicillium olsonii.* The taxonomic position of these strains within the genus *Penicillium* was investigated, resulting in the discovery of a new *Penicillium* species, described here as *P. salamii*. *Penicillium nalgiovense* and *P. salamii*, predominated on the meat surface. *P. salamii* was able to colonise salami effectively and may serve as a potential fungal starter, contributing to flavour development and protection against spoilage or pathogenic fungi.	[49]
14	Italy	This study analysed fungi on Culatello in three processing plants. Xerotolerant and xerophilic species (*Aspergillus* and *Penicillium*) dominated both meat and air. Moulds causing visual defects appeared early but declined, while ochratoxin-producing species were rare, posing minimal risk.	[50]
15	Japan	Dry-aged beef in Hokkaido developed surface moulds (*Mucor flavus*, *Helicostylum pulchrum*, *Penicillium*, *Debaryomyces*), which enhanced the aroma without affecting meat quality or fatty acid composition, except for a slightly higher C17:0 content.	[51]
16	Korea	During 40–60 days of dry-aging beef under controlled conditions, total bacteria and lactic acid bacteria increased, while foodborne pathogens and coliforms were absent. Early-stage potentially harmful yeasts/moulds (*Candida*, *Cladosporium*, *Rhodotorula*) disappeared over time, whereas *Penicillium camemberti* and *Debaryomyces hansenii* increased, suggesting fungi contribute to flavour and overall quality.	[52]

Table S3. *(continued)*

No.	Country	Highlights	Reference
17	Norway	A survey of Norwegian dry-cured meats identified 264 fungal isolates from 161 samples, representing 20 species across four genera. *Penicillium* dominated (88.3%), with *P. nalgiovense* the most frequent (38%), followed by *P. solitum* (13%) and *P. commune* (10%). *Cladosporium* and *Eurotium* were minor contributors. Smoking increased *P. nalgiovense* while reducing other species. *P. nalgiovense* can produce penicillin, posing a potential risk for allergic consumers.	[58]
18	Norway	A study of smoked hams and unsmoked dry-cured lamb analysed fungal contamination in the production process. From 642 samples, 901 fungal isolates were obtained, with yeasts dominating meat surfaces and moulds appearing mainly in late ripening. Over 39 mould species were identified, mostly *Penicillium*, especially *P. nalgiovense*, which can produce penicillin. Key contamination sources were improper pressing, poor air quality, and activities in sorting areas. Recommendations include reducing cracks, improving air circulation, and controlling airborne spores.	[59]
19	Portugal	A survey of Portuguese fresh and dry-cured meats analysed samples for ochratoxin A and aflatoxin B1, as well as the fungi responsible. A total of 630 fungal isolates were identified, with *Penicillium* as the dominant genus (66%). *Penicillium nordicum* and *Aspergillus westerdijkiae* were rare. OTA was detected in 40% of fresh pork, 43% of long-ripened pork legs, and 18% of shoulder hams, but not in goat or sheep products. AFB1 was not detected, indicating it is not a risk in these meats.	[13]
20	Slovakia	This study examined mould contamination in pork, beef, salami emulsions, and five types of fermented raw meats. The most common genera were *Penicillium*, *Acremonium*, *Mucor*, *Cladosporium*, and *Aspergillus*. Spices added during production, especially black pepper, nutmeg, garlic powder, and crushed caraway, showed the highest mould counts. Contamination was seasonal, with higher levels in summer.	[65]
21	Slovenia	The study focused on fungi on Slovenian dry-cured meats. *Penicillium* was the dominant genus, with eight species identified. *P. nordicum* was frequent, *P. nalgiovense* rare, and a new species, *P. "milanense"*, appeared in 21 items. *Aspergillus versicolor* was also detected, but it was less common. Several species, including *P. nordicum*, *P. brevicompactum*, *P. chrysogenum*, and *A. versicolor*, are mycotoxin producers and undesirable on meat.	[66]
22	Spain	*Penicillium* dominated the fungal population on Iberian hams during ripening, including *P. commune*, *P. chrysogenum*, *P. aurantiogriseum*, *P. expansum*, and *P. echinulatum*. Most strains were toxigenic, and toxicity increased over time. Non-toxigenic *P. chrysogenum* strains were identified as potential starter cultures to reduce health risks.	[67]
23	Spain	In dry-cured hams, *Penicillium* was the predominant fungal genus, especially in the ageing stage. Of 74 identified strains, 59 were *Penicillium*, representing 16 species. The most abundant were *P. commune* (24 strains) and *P. chrysogenum* (13 strains). About 34% of the strains produced cyclopiazonic acid, with *P. commune* and *P. polonicum* being highly toxigenic. Ochratoxin A production was less frequent (9.5%) and occurred in *P. chrysogenum*, *P. commune*, *P. polonicum*, and *P. verrucosum*.	[68]
24	Turkey	*Penicillium* was among the primary fungi detected in the air of meat plants, accounting for 23.7% of the isolates. Other dominant genera were *Mucor* and *Rhizopus*, while *Aspergillus* was less common.	[70]

Table S4. Overview of fungal contamination, focused on *Aspergillus* spp. and *Penicillium* spp., reported in retail meat markets across 14 studies (1990-2025).

No.	Country	Highlights	Reference
1	Austria / Austria and Italy	The study analysed samples of Speck from North and South Tyrol to identify the associated fungi. Among the isolated species, *Penicillium* was predominant. *Penicillium solitum* (together with *Eurotium rubrum*) dominated all Speck types and parts, and eight other *Penicillium* species were relatively frequent. Some species, such as *P. verrucosum*, *P. canescens*, and *P. commune*, are known to be potentially mycotoxigenic.	[26]
2	Croatia	The study investigated surface moulds on Croatian prosciuttos and fermented sausages from different regions and technologies and their relation to aflatoxin B1 and ochratoxin A contamination. *Penicillium* species dominated (79%), while *Aspergillus* (11%) and *Eurotium* (7%) were less frequent. Ochratoxin A contamination occurred in 14% of samples, and aflatoxin B1 in 8%. The detection of aflatoxin B1 in the absence of toxigenic moulds suggested contamination from spices or carry-over effects.	[15]
3	Egypt	Egyptian luncheon meat was frequently contaminated with moulds and yeasts, mainly *Aspergillus niger*, *A. flavus*, and *Penicillium chrysogenum*. *A. flavus* accounted for 10% of isolates. Out of 50 samples, seven contained aflatoxin B1 or B1 and G1, while other aflatoxins were absent. Some samples had high *A. flavus* counts but no detectable aflatoxins.	[35]
4	Egypt	In Egyptian basterma, total mould counts ranged from 10^3–10^6 CFU/g in summer and 10^2–10^5 CFU/g in winter, with *Aspergillus*, *Penicillium*, *Mucor*, *Rhizopus*, *Fusarium*, and *Cladosporium* being the most common genera. Aflatoxin B1 was detected in basterma and its spice components. Gamma irradiation effectively reduced contamination.	[36]
5	Egypt	A study on buffalo and cattle meat and edible offal found higher mould contamination in cattle than in buffalo samples. The neck muscles were the most contaminated, followed by the kidneys, liver, masseter muscles, and round meat. *Aspergillus* was the dominant genus, with species including *A. niger*, *A. flavus*, *A. fumigatus*, *A. ochraceus*, *A. parasiticus*, and *A. terreus*.	[38]
6	Egypt	This study analysed frozen beef samples from retail stores in Qena, Egypt, to identify and characterise fungal contamination. Moulds were found in 40% of samples, and yeasts in 60%. The dominant mould genera were *Aspergillus*, *Penicillium*, and *Cladosporium*. The findings emphasise the potential public health risks associated with fungal contamination of meat.	[39]
7	Egypt	In Mansoura, Egypt, meat samples, including sausages, beef burgers, minced meat, luncheon, hot dogs, and canned meat, were analysed for mould and aflatoxins. Moulds were found in all raw products and in 15–30% of heat-treated items, with *Aspergillus* and *Penicillium* being the most common genera. Heat-treated products had lower mould counts.	[40]

Table S4. *(continued)*

No.	Country	Highlights	Reference
8	Greece	In traditional Greek sausages, *Penicillium* species were found in 90.8% of visibly mouldy samples. The most common species were *P. solitum*, *P. nalgiovense*, and *P. commune*, which together comprised 60.6% of isolates. *P. nalgiovense* and *P. olsonii* were shown to produce penicillin, with penicillin production by *P. olsonii* reported for the first time.	[41]
9	Italy	**Already mentioned**	[26]
10	Nigeria	A survey of meat from Oyo State, Nigeria found contamination with *Aspergillus*, *Penicillium*, *Alternaria*, *Cladosporium*, *Fusarium*, *Neurospora*, *Rhizopus*, and yeasts, with *Aspergillus* being predominant. Potentially toxigenic fungi accounted for 38% of isolates. Aflatoxins B1, B2, G1, and G2 were detected in all samples, with kidney samples showing exceptionally high levels. .	[54]
11	Nigeria	A study of dried beef in Ekiti State, Nigeria found contamination with several filamentous fungi, including *Aspergillus* (43%), *Rhizopus* (42%), *Penicillium* (3%), *Fusarium* (2%), *Cladosporium* (2%), and *Alternaria* (1%). Among *Aspergillus* species, *A. flavus*, *A. niger*, and *A. fumigatus* were the most common, with *A. flavus* carrying genes for aflatoxin production.	[57]
12	Saudi Arabia	A study of canned meat in Saudi Arabia found *Aspergillus* and *Penicillium* in over 70% of 13 samples. Phylogenetic analysis of *Penicillium* isolates revealed four distinct groups.	[63]
13	Saudi Arabia	**Already mentioned**	[38]
14	Slovakia	**Already mentioned**	[65]

Table S5. Overview on other isolated *Aspergillus* spp. from samples in slaughterhouses, at meat processing stage, and markets (1990-2025).

Fungi Species	Location/Stage	Sort	Sample	Country
Aspergillus spp. **(including :** *Aspergillus aculeatus* *Aspergillus aeneus* *Aspergillus alabamensis* *Aspergillus alliaceus* *Aspergillus amstelodami* *Aspergillus caesiellus* *Aspergillus candidus* *Aspergillus carneus* *Aspergillus chevalieri* *Aspergillus cristatus* *Aspergillus cvjetkovicii* *Aspergillus flavofurcatis* *Aspergillus glaucus* *Aspergillus gracilis* *Aspergillus melleus* *Aspergillus montevidensis* *Aspergillus oryzae* *Aspergillus penicillioides* *Aspergillus proliferans* *Aspergillus pseudoglaucus* *Aspergillus repens* *Aspergillus ruber* *Aspergillus taichungensis* *Aspergillus tonophilus* *Aspergillus unguis* *Aspergillus ustus* **Non-specified** *Aspergillus spp.*	**Slaughterhouse**	Camel	Liver, lung, rumen, intestine, and head muscle	Egypt [37]
		Cattle	Air	Egypt [34]
			Floor	Serbia [64]
			Water, floor, wall, and beef carcass	Egypt [34]
		Sheep	Liver, lung, rumen, intestine, and head muscle	Egypt [37]
			Meat swabs	Iraq [44]
		Swine	Facilities and Surfaces	Korea [53]
		Poultry	Air	Austria [27] Italy [47]; Pakistan [60]; Portugal [61]
			Lungs	Brazil [30]
			Non-defined	Sweden [69]
		Non-defined	Houseflies	Iraq [45]
			Non-defined	India [42]
			Room, equipment, and tool	Saudi Arabia [62]
	**Meat processing**	Lamb/pork	Dry-cured meat production facility (hams, Fenalår, environment, air)	Norway [59]
		Mixed	Pork leg (14- and 20-month curing periods), pork shoulder, goat, and sheep	Portugal [13]
			Liver pâté from one plant (autumn)	Denmark [33]
			Sausage/ham	Argentina [25]; Brazil [29;28]; Croatia [32]; Egypt [12]; Italy [11;48;50]; Norway [58]; Spain [68]
		Pork or beef	Fermented sausage from one plant (autumn)	Denmark [33]
		Pork	Harbin dry sausages during fermentation	China [31]
		Non-defined	Air samples across 3 meat processing plants (summer)	Turkey [70]
	**Market**	Beef	Basterma paste (coat), meat, and coat and meat	Egypt [36]
			Round muscle, neck muscles, masseter muscles, liver, and kidney	Saudi Arabia [38]
		Bufallo	Round muscle, neck muscles, masseter muscles, liver, and kidney	Egypt [38]
		Non-defined	Dried meat sample collected from 5 major markets	Nigeria [54]
			Dry Meat	Nigeria [57]
			Croatian traditional dry-cured meat products	Croatia [15]
			Frozen meat samples on PDA and DRBC	Egypt [39]
			Sausage, beef burger, and minced meat (raw meat products), and luncheon, hot dog, and canned meat (heat-treated meat products)	Egypt [40]

Table S6. Overview on other isolated *Penicillium* spp. from samples in slaughterhouses, at meat processing stage, and markets (1990-2025).DRBC: Dichloran Rose Bengal Chloramphenicol agar; PDA: Potato Dextrose Agar

Fungi Species	Local	Sort	Positive Sample(s)	Country
Penicillium spp. **(including:** ***Penicillium aethopicum, Penicillium atramentosum, Penicillium camemberti, Penicillium carneum, Penicillium caseifulvum Penicillium corylophilum, Penicillium cavernicola, Penicillium citreonigrum, Penicillium corylophilum, Penicillium cvjetkovicii, Penicillium decaturense, Penicillium echinulatum, Penicillium fagi, Penicillium fellutanum, Penicillium frequentans, Penicillium glandicola, Penicillium hirsutum, Penicillium hordei, Penicillium implicatum, Penicillium janczewskii, Penicillium jensenii, Penicillium lanoso-coeruleum, Penicillium lanoso-griseum, Penicillium lanosum, Penicillium melenii, Penicillium miczynskii, Penicillium minioluteum, Penicillium milanense (ined.), Penicillium nalgiovense, Penicillium notatum Penicillium olsonii, Penicillium oxalicum, Penicillium phoeniceum, Penicillium pinophilum, Penicillium purpurogenum, Penicillium puberulum, Penicillium raistrickii Penicillium restrictum, Penicillium salami, Penicillium section Camembertiorum,***	**Slaughterhouse**	Camel	Liver, lung, rumen, intestine, and head muscle	Egypt [37]
		Cattle	Air	Egypt [34]; Nigeria [56]; Portugal [61]; Serbia [64]
			Beef carcass	Egypt [34]; Nigeria [56]
			Floor	Egypt [34]; Serbia [64]
			Slaughter ground Scrapings	Nigeria [56]
			Wall	Egypt [34]
			Water	Egypt [34]
		Cattle/poultry	Air	Pakistan [60]
		Poultry	Air	Austria [27]; Italy [47]; Portugal [61]
			Lungs	Brazil [30]
			Non-defined	Sweden [69]
		Sheep	Liver, lung, rumen, intestine, and head muscle	Egypt [37]
		Swine	Facilities and Surfaces	Korea [53]
		Swine/Beef	Air	Portugal [61]
		Non-defined	Air	Iraq [43]
			Surfaces	Nigeria [55]
			Tool	Saudi Arabia [62]
			Non-defined	India [42]
	**Meat processing**	Beef	Initial point of dry aging (beef)	Argentina [24]
		Beef or pork	Fermented sausage from two plants (spring and autumn)	Denmark [33]
		Lamb	Dry-cured meat production facility (hams, Fenalår, environment, air)	Norway [59]
		Mixed	Pork leg (14- and 20-month curing periods), pork shoulder, goat, and sheep	Portugal [13]
			Spices: Prime beef rump steak (summer and winter), industrially prepared deboned ham (summer), pork flank without skin (winter), bacon without skin (spring), crushed caraway (summer and winter), garlic powder (summer), milled black pepper (summer, winter, and spring), nutmeg (winter and spring), and sugar (spring)	Slovak Republic [65]
			Ham portion, ham muscle and air	Italy [46]
			Liver pâté from two plants (spring and autumn)	Denmark [33]
			Sausage/ham	Argentina [25]; Brazil [28;29]; Croatia [32] Egypt [12]; Italy [11;49;48;50]; Japan [51]; Korea [52]; Norway [58]; Slovenia [66]; Spain [67;68]

Penicillium section brevicompacta, Penicillium solitum, Penicillium spinulosum, Penicillium thymicola, Penicillium tricolor, Penicillium vindication, Penicillium waksmanii, Penicillium canescens, Non-specified Penicillium spp.
